# Lipid-mediated prestin organization in outer hair cell membranes and its implications in sound amplification

**DOI:** 10.1038/s41467-022-34596-9

**Published:** 2022-11-12

**Authors:** Sepehr Dehghani-Ghahnaviyeh, Zhiyu Zhao, Emad Tajkhorshid

**Affiliations:** Theoretical and Computational Biophysics Group, NIH Center for Macromolecular Modeling and Bioinformatics, Beckman Institute for Advanced Science and Technology, Department of Biochemistry, and Center for Biophysics and Quantitative Biology, University of Illinois at Urbana-Champaign, Urbana, IL USA

**Keywords:** Computational biophysics, Membrane biophysics, Motor protein function

## Abstract

Prestin is a high-density motor protein in the outer hair cells (OHCs), whose conformational response to acoustic signals alters the shape of the cell, thereby playing a major role in sound amplification by the cochlea. Despite recent structures, prestin’s intimate interactions with the membrane, which are central to its function remained unresolved. Here, employing a large set (collectively, more than 0.5 ms) of coarse-grained molecular dynamics simulations, we demonstrate the impact of prestin’s lipid-protein interactions on its organization at densities relevant to the OHCs and its effectiveness in reshaping OHCs. Prestin causes anisotropic membrane deformation, which mediates a preferential membrane organization of prestin where deformation patterns by neighboring copies are aligned constructively. The resulting reduced membrane rigidity is hypothesized to maximize the impact of prestin on OHC reshaping. These results demonstrate a clear case of protein-protein cooperative communication in membrane, purely mediated by interactions with lipids.

## Abstract (web summary)

Prestin, a motor protein, plays a major role in sound amplification. Using molecular dynamics simulations, the authors show that prestin causes membrane deformation patterns thereby achieving a particular lipid-mediated alignment in the membrane.

## Introduction

Hearing is a major sensory mechanism evolved to enable higher organisms to detect auditory cues from their environment^1,2^. Species such as humans and other mammals are equipped with a complex auditory system, composed of several organs that are involved in different steps of the hearing process. Many of the complexities engineered into the hearing system have been evolved for distinguishing between different frequencies^1,2^. A key aspect of hearing, however, is sound amplification within a spiral-shaped organ in the inner ear known as the cochlea, without which the hearing system is dysfunctional^3^. Cochlear amplification relies on specialized cells known as the outer hair cells (OHCs) and their unique piezoelectric character^4,5^. Modulations in the membrane potential of the OHCs result in their mechanical response in the form of vibration (a property termed “electromotility”)^4,5^. This vibration is synchronized to the sound signal and provides mechanical amplification by feeding back into the traveling wave^4–6^. The source of electromotility in the OHCs is a motor membrane protein, called prestin, which generates somatic forces and alters the structure of the OHCs as a result of its voltage-dependent conformational changes^7–9^. Dysfunction of prestin or other elements in the OHCs, e.g., by excessive noise or aging, can result in a reduction of sound sensitivity or even total deafness^10–12^.

Prestin is a member of the anionic transporter superfamily, SLC26A5^13,14^, and similar to other members of the superfamily, it binds to anions (e.g., Cl^−^)^15^. However, unlike the other members, which function as transport proteins, prestin acts as a motor protein. Despite its discovery and biochemical characterization for decades^8,9,16,17^, the structure of prestin was resolved only recently^18,19^. The cryo-EM models of prestin revealed a homodimeric architecture with a two-fold symmetry^18,19^. The transmembrane (TM) region of each protomer is composed of two domains, the core domain (residues 80-202 and 338-432) and the gate domain (residues 209-315 and 437-497), forming an anion binding site at their interface^18–21^.

The structure of prestin has been captured with bound Cl^−^ ions (agonist), as well as with other negatively charged ligands, e.g., the inhibitor salicylate^18,19^. In the agonist (Cl^−^)- or inhibitor-bound structures, the core and gate domains exhibit different relative arrangements, generating two conformational/functional states coined as contracted and expanded, with a smaller protein cross-sectional area in the former^18,19^. The transition between these two conformations is hypothesized to be the source of prestin-mediated vibration of the OHCs, and, therefore, key to sound amplification in the cochlea^18^.

Freeze-fracture electron microscopy has shown a high density of prestin on the surface of the OHCs, estimating a separation of only ~200 Å between prestin dimers in the cellular membrane^22^. The structural changes of the OHCs in response to prestin’s activation can therefore be substantial^15,22^. The packing ensures that even small conformational changes of individual prestin dimers will add up to sufficiently large structural changes of the whole cell, and, therefore, to effective sound amplification^22,23^. Given its high cellular density, it is important to understand the organization of prestin dimers within the membrane, which might in turn affect their effectiveness and even cooperativity.

As described in previous studies, the interaction between the prestin and its embedding membrane is crucial for its role in sound amplification^24–30^. Nevertheless, and despite the availability of atomic-resolution structures^18,19^, the details of prestin’s interactions with lipids and the membrane remain largely elusive.

Lipid-protein interactions have been the subject of numerous studies, as lipids play an active role in modulating the structure and function of membrane proteins^30–33^. Owing to their high spatial and temporal resolutions and their complementarity to experimental results, molecular dynamics (MD) simulations have played a major role in our current understanding of lipid-protein interactions in near-native membranes^33–39^. In order to improve sampling of lipid-protein interactions, especially in heterogeneous membranes, many MD studies have used coarse-grained (CG) representations, e.g., using MARTINI, a widely used CG method for lipidic structures^40,41^. MARTINI CG simulations of membranes and membrane proteins have contributed significantly to our understanding of how membrane proteins modulate their lipidic environments, both at the distribution and structural levels^42,43^.

Here, we report an extensive set of MD simulations performed to characterize the structural effects of prestin on the membrane. Using different simulation designs, we also ask the question whether these effect might in turn modulate prestin’s organization in the membrane at densities representing its concentration in the OHCs. The simulations capture consistently a profound effect of prestin in deforming the membrane, in a particular pattern caused by anisotropically elevating or depressing lipids at its different flanks, an effect that extends over a long range of ten nanometers away from the protein. More importantly, multiple-prestin simulations show that the patterns of membrane deformation induced by the neighboring proteins is used to align their orientations. This through-lipid communication and entanglement between the proteins provide an energetically favorable membrane organization with a lower bending modulus, which can offer a larger cellular response, and therefore more effective in producing the mechanical role of prestin in the OHCs.

## Results and discussion

The simulations in each part were performed on systems either with contracted or expanded prestin dimers. The figures and data presented in the main text describe the results for the contracted conformation; corresponding results for the expanded conformation are provided as Supplementary information.

### Prestin induces anisotropic deformation in the membrane

**Fig. 1:**
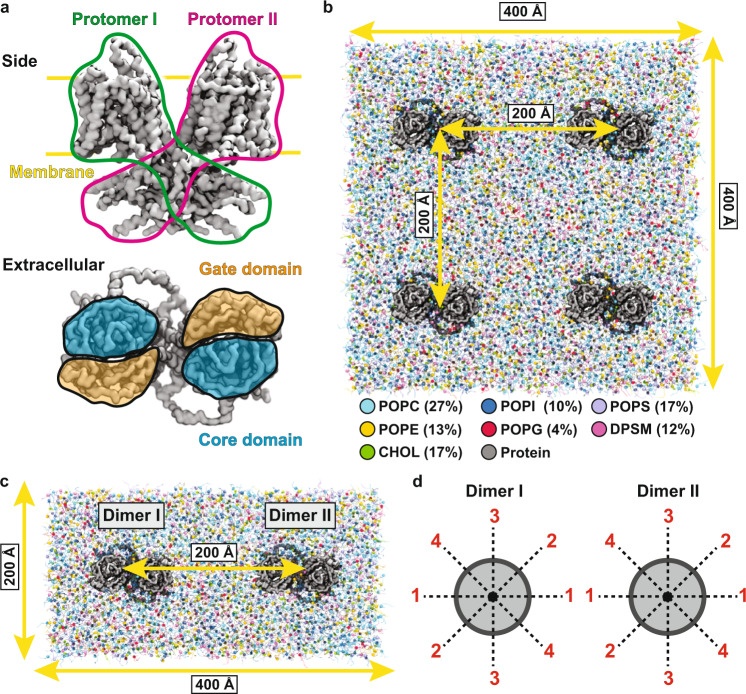
Prestin simulation systems. **a** MARTINI-based CG model of prestin from the side and extracellular views. In the side view, the two protomers are outlined in green and magenta, respectively, and the approximate position of the membrane is marked with yellow lines. In the extracellular view, the core and gate domains are colored in orange and blue, respectively. **b** Simulation system to probe lipid-protein interactions, including four prestin dimers, separated by 200 Å, and embedded in a square membrane patch of side length 400 Å. The composition of the membrane is shown in the legend, with lipid types represented with different colors. **c** Simulation system to study protein-protein cross-talks. Each system includes two prestin dimers at a 200-Å separation, embedded in a 400 × 200 Å^2^ bilayer, and with different relative orientations **d**. In each system, the two dimers are rotated around the membrane normal (*z* axis) in 45° increments. Given the two-fold symmetry of the protein, combinations of four different orientations per protein dimer (0°, 45°, 90°, and 135°, labeled as 1–4) cover all relative orientations of the two proteins at 45° intervals. Identical orientations are numbered the same, e.g., 45° and 225° are both labeled 2.

**Fig. 2:**
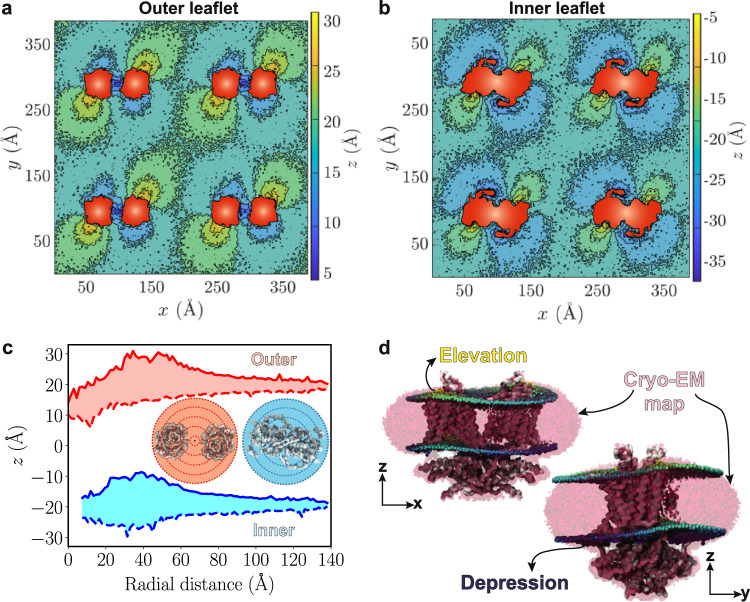
Prestin-induced, anisotropic membrane deformation. **a** and **b** 2D histograms of the *z* positions of lipid headgroups for outer and inner leaflets. Both leaflets are viewed from the extracellular side. Histograms are constructed from the last 5 μs of 20-μs trajectories. The cross-sectional areas of the prestin dimers in each leaflet are colored in red. **c** Range of *z* values of lipid headgroups in radial sections (drawn in the inset) around the protein, red for the outer and blue for the inner leaflet. The maximum elevation values are represented with solid and minimum values with dashed lines, whereas the range of observed *z* values is shown in light red and blue. The blue curves do not start from zero, since in the inner leaflet there are no lipids in that region. Source data are provided as a Source Data file. **d** Agreement between the lipid density in the cryo-EM map and the phospholipid distribution from the simulations, averaged over the last 5 μs of one of the trajectories. The cryo-EM density is represented as a red mesh. The coloring of headgroups is based on their *z* values, with highest and lowest *z* shown in yellow and blue, respectively. Elevated and depressed regions of the membrane form MD simulations overlap with corresponding areas in the cryo-EM map. For a rotating animation showing this comparison from different views, see Supplementary Movie 1.

To investigate the effect of prestin on the surrounding membrane, we probed the structure of a membrane composed of 4 prestin dimers (separated by 200 Å) embedded in a lipid mixture (quad-prestin) over 20 *μ*s of CG MD simulation (Fig. 1b). The membrane structure was analyzed using the *z* positions of lipid headgroups in small bins to provide a metric with sufficiently detailed spatial resolution. The constructed 2D histograms indicate non-uniform deformation of the membrane by prestin, manifested by elevation or depression of the lipids flanking different sides of the protein, in both the inner and outer leaflets (Fig. 2a, b). The membrane was centered at *z* = 0, with the phospholipid headgroups of the outer and inner leaflets initially positioned at approximately *z* = +20 and *z* = −20 Å, respectively. During the simulation, the phospholipid headgroups displace to *z* values ranging from +5 to +30 Å in the outer leaflet, and from −5 to −30 Å in the inner leaflet, corresponding to displacements as large as 15 Å from their initial positions in each leaflet (Fig. 2a, b).

The depressed lipids in the outer leaflet are primarily associated with the core domains, particularly in the region between the two prestin protomers, highlighted by *z* values as low as *z* = 5 Å (Fig. 2a and Supplementary Fig. 1a). Notably, the region between the two protomers was initially free of lipids but becomes completely filled during the simulation in all four protein copies (Supplementary Fig. 1a). In contrast to the depressed membrane regions, elevated lipids in the outer leaflet arise in the vicinity of the gate domains of prestin where lipid elevations as large as *z* = 30 Å are observed.

Similar to the outer leaflet, the pattern of membrane deformation in the inner leaflet can be characterized by lipid elevation in the proximity of the gate domains and lipid depression in the vicinity of the core domains (Fig. 2b and Supplementary Fig. 1b). In the inner leaflet, the elevated lipids reach *z* values of about −5 Å, whereas in the depressed region lipids sink to as low as *z* = −30 Å (Fig. 2b). Similar membrane deformation patterns were observed for both the outer and inner leaflets in the case of the expanded prestin conformation (Supplementary Fig. 2a, b).

In order to quantify the range over which prestin deforms the membrane, we defined radial regions around the protein, and for each region, the minimum and maximum *z* values of lipids were recorded. The analysis clearly establishes the long range of prestin-induced membrane deformation, extending as long as 10 nm away from the protein (Fig. 2a-c and Supplementary Fig. 2a, b). Membrane deformation around the protein follows the two-fold symmetry of prestin’s dimeric structures (Fig. 2a, b and Supplementary Fig. 2a, b), and deformation patterns of individual prestin dimers are highly consistent, supporting the convergence of the simulations (Fig. 2a, b and Supplementary Fig. 2a, b).

The deformation patterns captured here indicate a strong, but heterogeneous membrane response to prestin and are in close agreement with the observed lipid densities around the protein in cryo-EM studies of nanodisc-embedded prestin^18,19^. Overlapping the cryo-EM map for prestin structure (e.g., in the contracted conformation (PDB ID: 7LGW [10.2210/pdb7LGW/pdb])^18^) and the positions of phospholipid headgroups from the simulations, a close agreement between the computational and experimental results is evident (Fig. 2d and Supplementary Movie 1).

Apart from deformation, membrane thickness, area, and atomic mass distribution of lipids and water were monitored during the last 5 μs of the simulations, assuring the stability and healthiness of the simulated membrane systems (Supplementary Fig. 3a–c).

### Prestin alignment mediated by membrane deformation

**Fig. 3:**
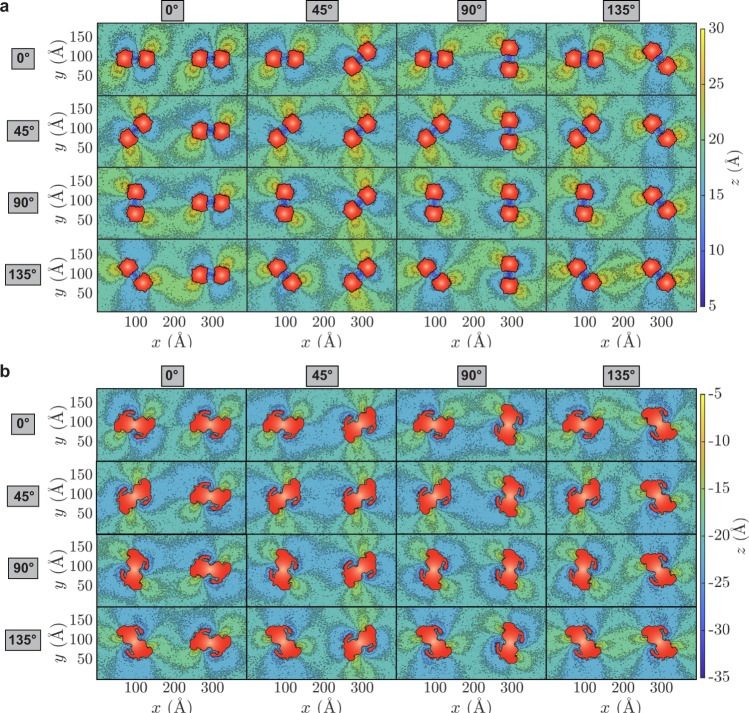
Constructive or destructive interference of deformation patterns induced by prestin’s different orientations. Heatmaps of phospholipid height in the outer (**a**) and inner (**b**) leaflets are calculated using the procedure described in Fig. 2. The prestin dimers are placed at (*x*, *y*) = (100, 100) Å and (300, 100) Å, respectively. The orientation of Dimer I (angle with respect to the *x* axis) is specified on the left, and for Dimer II on the top of each panel. Given the symmetry relation between prestin’s protomers, four angles (0°, 45°, 90°, and 135°) for each prestin dimer are sufficient to cover all possible orientations (at 45° intervals), resulting in 16 different combinations of two dimers. The protein cross-sectional area in each leaflet is drawn in red.

To characterize membrane deformation patterns produced by different prestin arrangements, we designed an array of simulation systems each with two prestin dimers embedded in a rectangular membrane at a fixed prestin-prestin distance but with different relative orientations (Fig. 3). Membrane deformation patterns were monitored by constructing lipid height heatmaps (Fig. 3a, b for contracted prestin, and Supplementary Fig. 4a, b for expanded prestin). The heatmaps immediately highlight an important relationship of membrane deformation patterns, namely the significant overlap between the patterns induced by neighboring prestin dimers, indicating that the proteins might indirectly influence each other’s orientation through their effects on the membrane. For example, the configuration with two prestin dimers at 0° ([0°, 0°]; top left in Fig. 3a, b) generates non-matching, destructive deformation patterns (Fig. 3a, b). However, as the prestin dimers are rotated in other simulation systems, the membrane deformation patterns start to align with each other, thereby creating constructive interference profiles, e.g., in the [45°, 45°] configuration (Fig. 3a, b). Interestingly, when the membrane deformation patterns are constructively aligned, e.g., in the [45^∘^, 45^∘^] configuration, the structural effect of prestin on the membrane seems to be propagating over a longer range (Fig. 3a, b). The membrane deformation patterns for expanded prestin dimers are similar to those obtained from contracted systems, but generally with a smaller propagation range (Fig. 3a, b and Supplementary Fig. 4a, b).

**Fig. 4:**
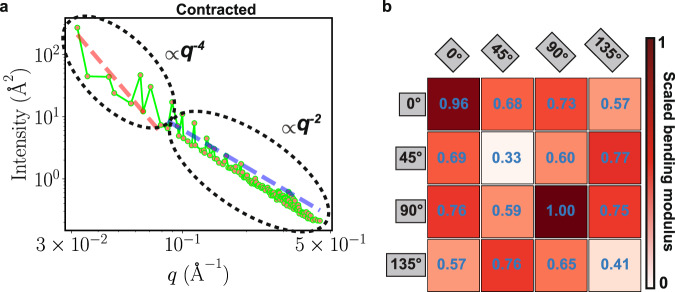
Membrane bending moduli calculated for different arrangements of contracted prestin. **a** Intensity, ∣*h*(*q*)∣^2^ plotted vs. *q*, wavenumber, for the system with two prestin dimers both at 135° ([135°, 135°]). The plot is in log-log format and contains two regions: (1) free-undulation (fitted to the *q*^−4^ line shown in red) and protrusion (fitted to the *q*^−2^ line shown in blue). The fit to the free-undulation region is used to calculate the bending modulus. Source data are provided as a Source Data file. **b** Bending moduli for the membranes with two prestin dimers in different relative orientations (Fig. 3), normalized by the maximum value for the [90°, 90°] system. The orientation of Dimer 1, placed at (*x*, *y*) = (100, 100) Å is shown on the left, and for Dimer II, placed at (*x*, *y*) = (300, 100) Å, on the top of the table. The color shade in each box also represents the strength of the scaled bending modulus, and defined in the scale bar. The minimum bending moduli are captured when the two prestin dimers are both at either 45° or 135°.

To more quantitatively estimate and compare the different deformation patterns caused by prestin’s orientation and their effect on the membrane structural response, the associated membrane stiffness was estimated by calculating the membrane bending modulus, which is directly proportional to membrane bending energy. The results show that when membrane deformation patterns are in line, the membrane bending modulus is minimal (Fig. 4a, b and Supplementary Table 1), suggesting that in these configurations the change in the cellular membrane shape in response to the mechanical activity of the protein might be maximal. In contrast, in cases with destructive interference between the deformation patterns, i.e., when depressed lipids around one prestin dimer face lipids elevated by a neighboring dimer, the membrane exhibits a larger degree of rigidity (Figs. 3a, b and 4a, b).

To ensure that the calculated bending moduli are not affected by largely deformed lipids in the close vicinity of the protein, we redid the *K* calculations after excluding lipids within the 7 Å of the protein for all systems. To further study the effect of local lipids in the calculations of the bending moduli, in one case, [135°, 135°], we compared the results of the calculations with three groups of lipids, separately: (1) all lipids included, (2) after exclusion of lipids within 7 Å of the protein, and, (3) after exclusion of lipids within 14 Å of the protein. As shown in Supplementary Fig. 5, despite minor differences, the three graphs, especially on a log scale, look identical, and the resulting bending moduli from the fits are nearly the same.

The lowest bending modulus (softest membrane) for contracted prestin simulations is obtained when the dimers are both at either 45^∘^ or 135^∘^. In these configurations, the depressed and elevated lipids surrounding reach their maximum propagation to fully extend between the two prestin dimers (Fig. 3a, b and Supplementary Fig. 4a, b). Note that systems with both prestin dimers either at 45° or at 135° correspond to the same relative configurations (due to the C2 symmetry in prestin’s dimer). For the same system, the highest bending modulus is obtained when the neighboring dimers are both at 90^∘^ ([90^∘^, 90^∘^]; Fig. 4a-b), where maximal mismatch between depressed and elevated lipids from the two dimers arises. Similar profiles are obtained for the simulations of expanded prestin (Supplementary Fig. 6a, b and Supplementary Table 1). The lowest bending moduli for both contracted and expanded prestin correspond to [45°, 45°] or [135°, 135°] orientations, although for the contracted system most bending moduli remain close to their lowest value (bright shades; Fig. 4b), whereas in expanded prestin they differ significantly (dark shades; Supplementary Fig. 6b). One might speculate that in its contracted form prestin is more free to alter its relative orientation, whereas in its expanded form it is more confined to a particular orientation.

The calculated membrane deformation patterns suggest that prestin dimers on the surface of OHCs might be organized with a preferred orientation of 45/135°, such that their elevated and depressed lipid regions align, thereby achieving the longest range and largest degree of membrane deformation (Fig. 3a, b and Supplementary Fig. 4a, b). Given the prestin density in the simulations, its favored relative orientations observed here are of direct relevance to the crowded arrangement of prestin in the OHCs. At these particular prestin arrangements, the membrane achieves the lowest bending modulus (softest membrane), which in turn assists the OHCs to fluctuate more in response to sound signal and therefore, generate a more pronounced sound amplification.

As far as membrane rigidity is concerned, biology could have chosen other means, e.g., changing the lipid composition of the cell, to achieve similar effects. However, we might speculate that the preferred prestin arrangements observed in our study might be of additional ramifications that are still unresolved. While, to the best of our knowledge, there are currently no experimental data on either the relative orientations of prestin dimers in the OHCs or their effect on the membrane’s mechanical properties, experimental methods such as single-molecule fluorescence microscopy, which has been used previously to monitor the rotational motions of macromolecular systems^44,45^, can potentially provide insight into the orientation of prestin in OHC membranes.

**Fig. 5:**
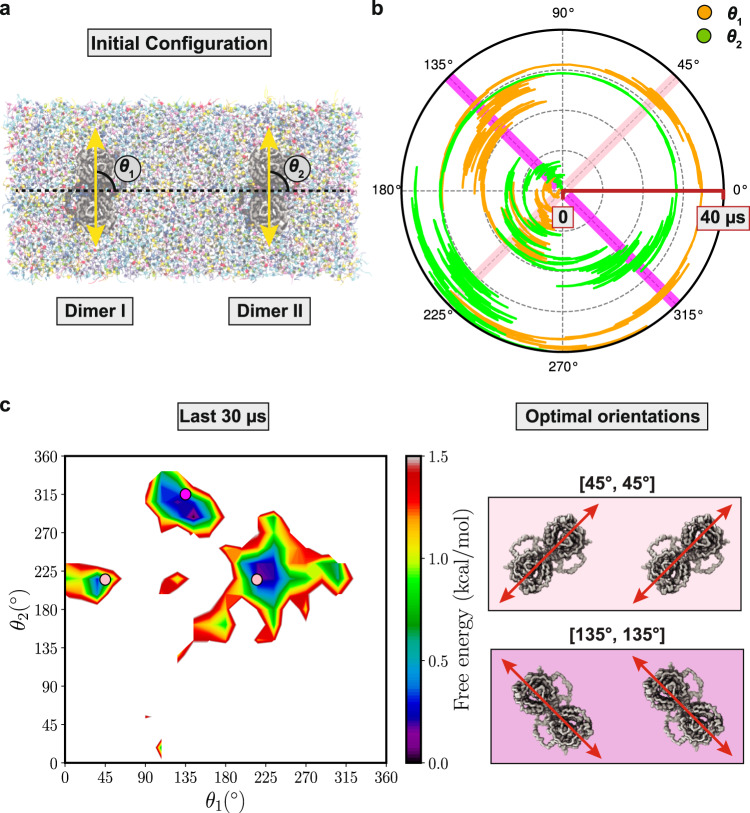
Prestin-prestin communication through lipids. **a** Initial system setup with two contracted prestin dimers at 90° [90°, 90°]. The center of mass of each dimer is restrained, but they are allowed to rotate freely. Orientation of the two prestin dimers are denoted as *θ*_1_ and *θ*_2_, respectively. **b** Time series of *θ*_1_ (orange) and *θ*_2_ (green) during the 40 *μ*s simulation. The radial direction in the polar plot represents the time from 0 to 40 μs. See also Supplementary Movie 2. Source data are provided as a Source Data file. **c** Free energy landscape in *θ*_1_/*θ*_2_ space, constructed from the last 30 μs of the trajectory. The landscape consists of three main minima. The pink and magenta circles correspond to [45°, 45°] and [135°, 135°] prestin-prestin configurations, respectively, also highlighted in **b** by diagonal bars.

To further examine the preferred prestin-prestin configuration in the membrane, we designed another simulation in which neighboring prestin dimers were allowed to freely rotate (Fig. 5a). The orientations of the two dimers (*θ*_1_ and *θ*_2_) were then monitored during a 40-μs simulation (Fig. 5b). The time evolution of the angles confirms our conclusion regarding the preferred orientations of prestin in the membrane: either at ~45° (same as 225°) or at ~ 135° (same as 315°) (Fig. 5b and Supplementary Movie 2). Furthermore, the neighboring dimers seem to sense and follow each other’s orientation, in that when one of them changes its orientation, the other follows. For instance, at *t* = ~20 μs, when one dimer alters its orientation from ~225° to ~135° (orange trace in Fig. 5b and Supplementary Movie 2), the second dimer (green trace) follows immediately and changes its orientation from ~225° to ~315° (Fig. 5b). Such membrane-mediated communication between prestin dimers is evident at several time points during the simulation (Fig. 5b and Supplementary Movie 2). The free energy landscape in *θ*_1_/*θ*_2_ phase space, constructed from the last 30 *μ*s of the simulation, reveals three different minima (Fig. 5c), all close to the optimal configurations predicted from our earlier simulations, i.e., corresponding to [45°, 45°] and [135°, 135°] configurations (Fig. 5c).

To verify that the preferred prestin arrangement are not affected by PBC, in another setup, we simulated nine prestin dimers distanced 200 Å from each other and placed in a lipid bilayer with 800 Å × 800 Å dimension (*nona-prestin*). This system setup contains a larger bilayer padding surrounding prestin dimers, assuring that PBC does not add any artifacts to the simulations. The centered dimer (which is surrounded by eight dimers) is free to rotate, while the other eight dimers are restrained to 45° orientation (Supplementary Fig. 7a). This new set contains four different simulations with different initial orientations for the free-rotating prestin (0°, 45°, 90°, and 135°), preventing any possible induced-bias from the initial arrangement (Supplementary Fig. 7a). Each system was simulated for 5 μs and the orientation of the free-rotating dimer was monitored and histogrammed over the last 4 μs of trajectories. The maximum population of the constructed histogram is located around 45°, the most favorable arrangement predicted previously, and therefore confirming that PBC does not induce any artifact in our conclusions (Supplementary Fig. 7b, c).

### Enrichment of POPI and CHOL around prestin

**Fig. 6:**
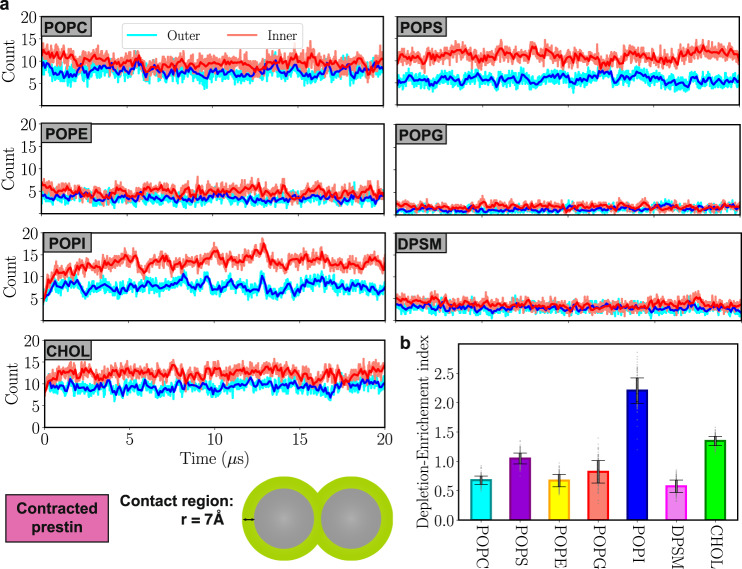
Enrichment/depletion of different lipids around contracted prestin. **a** Time series of the number of different lipid types within 7 Å of a prestin dimer, averaged over four dimers, in the outer (blue) and inner (red) leaflets. POPI lipids accumulate the most around prestin in the inner leaflet. **b** Depletion-Enrichment index of each lipid type (the ratio of the local and bulk fractions of the lipid). POPI and CHOL show the highest enrichment, whereas DPSM and POPE show the largest degree of depletion. Data points in each bar plot are shown as gray dots. Error bars are standard deviation of the data obtained from 2000 data points. Source data are provided as a Source Data file.

In addition to membrane deformation, we also observe differential enrichment or depletion of different lipid types around prestin (Fig. 6a). For CHOL, and particularly for POPI, the simulations record large increases in their local density around the protein (Depletion-Enrichment indices of 1.4 and 2.2, respectively; Fig. 6b). As shown in Fig. 6a, the initial numbers of 4 POPI and 8 CHOL lipids in the vicinity of prestin in each leaflet increase to 8-15 POPI and 10-15 CHOL per leaflet over the 20-*μ*s course of the simulation. In contrast, the number of POPC, POPE, POPG, and DPSM lipids in the vicinity of the protein in both the outer and inner leaflets slightly drops, mostly during the first 2 *μ*s (Depletion-Enrichment indices less than one; Fig. 6b). Despite being a charged lipid, the POPS count close to prestin remains relatively unaltered during the simulation (Depletion-Enrichment index of nearly one; Fig. 6b). Approximately similar counts and enrichment/depletion indices are obtained for expanded prestin simulations (Supplementary Fig. 8a, b).

**Fig. 7:**
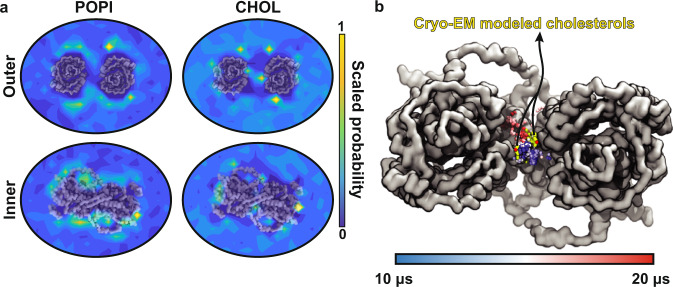
Accumulation of POPI and CHOL around prestin. **a** Heatmaps representing the distribution of POPI and CHOL over the last 5 μs of the quad-prestin trajectory in the contracted conformation. Both POPI and CHOL cluster closely to the core and gate domains of prestin. The region in between the two protomers contains two distinct CHOL binding sites but remains free of POPI during the simulation. **b** Comparison of cryo-EM and MD CHOL. The trajectory of one of the CHOL molecules in between the two protomers during the last 10 *μ*s of the simulation is shown in varying colors representing the time (scale bar). Only the hydroxy bead is shown for each frame. The cryo-EM CHOL models are shown in yellow. Source data are provided as a Source Data file.

To pinpoint high-density regions of POPI and CHOL around prestin, we calculated the 2D distribution of these lipids during the last 5 *μ*s of the trajectory for all four prestin dimers in the *quad-prestin* simulation. POPI accumulation is mostly observed around the core and gate domains, with higher densities around the latter in the outer leaflet, and around the former in the inner leaflet (Fig. 7a). In the case of CHOL, high-occupancy regions are observed more scattered around the protein (Fig. 7a). Of particular significance, two CHOL high-density regions are positioned symmetrically in between the two protomers in the outer leaflet (Fig. 7a), i.e., at the interface of the core and gate domains of each protomer (Fig. 7a). The MD trajectory shows that, once bound, these inter-dimeric CHOL molecules tend to stay in this site while sampling different conformations (see snapshots from *t* = 10–20 μs in Fig. 7b). Supporting these observations, the cryo-EM model of contracted prestin contains two symmetrically positioned CHOL molecules at this location between the two protomers^18^. Despite thermally driven fluctuations, the CHOL trajectory samples conformations similar to cryo-EM poses (Fig. 7b). Interestingly, the region between the two prestin protomers remains free of POPI throughout the simulation (Fig. 7a).

Clustering of PI and CHOL has also been reported for other anionic carriers. In a MD study of Band 3 (*aka* AE1 or SLC4A1), a Cl^−^/HCO${}_{3}^{-}$ exchanger in red blood cells and kidneys, annular lipids were found to be enriched in PI and CHOL^46^. Clustering of POPI and CHOL lipids close to prestin, reported in our study, might play a role in prestin’s conformational dynamics, as these lipids can bind membrane proteins through specific, often functionally important, binding sites^47–50^.

### Implications in hearing

Prestin is a high-density motor protein on the surface of the OHCs, whose conformational changes constitute the main mechanism of cochlear sound amplification in mammalian auditory systems. Recent advances in structural studies have characterized the protein in both active and inactive conformational states. However, the extent and nature of its interaction with the lipids and the ensuing membrane deformation by prestin, which are at the heart of its effect on the shape of the OHCs remained elusive.

**Fig. 8:**
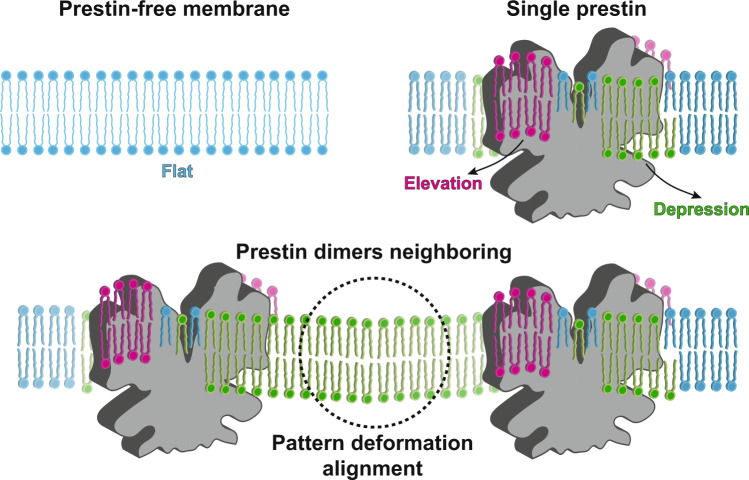
Membrane deformation patterns induced by a single or multiple-prestin dimers. A prestin-free membrane remains flat, as highlighted by the even height of its lipids (blue). Prestin deforms the membrane anisotropically by elevating or depressing lipids (magenta and green, respectively) in its vicinity. In a multi-protein arrangement, as in the OHCs, membrane deformation patterns of individual prestin dimers can align constructively, with matching elevated and depressed lipids. These favorable prestin-prestin configurations, which likely represent its preferred arrangement in the OHCs, offer a longer range of membrane deformation as well as a softer membrane with a greater extent of response to prestin’s mechanical role in sound amplification.

Here, using an extensive set of MARTINI CG MD simulations (collectively, 540 μs) we characterize the nature of membrane structural effects of prestin. The simulations reveal strong anisotropic membrane deformation patterns, manifested by regions with elevated or depressed lipids, induced by the gate and core domains of protein. These deformation patterns are long range and last for ~10 nm in both the outer and inner leaflets. The elevated and depressed regions are enriched in CHOL and POPI, suggesting possible effects on the function of prestin. The observed membrane deformation and its long range of propagation suggested possible, lipid-mediated communication between prestin dimers at surface densities observed in OHCs. This aspect was investigated by simulations involving pairs of prestin dimers with different relative orientations and quantifying their effects on membrane structure and rigidity. Both rotationally restrained and free-rotating simulations clearly highlight favorable orientations of neighboring prestin molecules that allow for maximal alignment of membrane deformation patterns (Fig. 8) and a reduction in the bending modulus of the membrane, thereby resulting in a more responsive membrane to mechanical forces caused by prestin. These structural and mechanical properties of the membrane are of high relevance to prestin’s biological role as the main element of cochlear sound amplification.

## Methods

### System setup

The prestin cryo-EM structures in contracted (Cl^−^-bound) and expanded (salicylate-bound) conformations were used as the starting models to construct initial all-atom (AA) models (Supplementary Fig. 9a–d)^18^. The bound ligands and modeled lipids were removed from the cryo-EM structures. Both protein structures lack a disordered region in the intracellular domain, dividing them into two polypeptide segments (residues 13-580 and 614–724, respectively). N-terminal ammonium and C-terminal carboxy caps were added to the first and last residues in each segment, respectively, employing the PSFGEN plugin of VMD^51^. All hydrogen atoms as well as the missing side chains were also added to the structures using PSFGEN.

The initial AA models were then converted to MARTINI CG models using the MARTINIZE protocol as described on the MARTINI website (http://www.cgmartini.nl/), including an elastic network restraining pairs within a 10-Å cutoff (Fig. 1a). The protein’s secondary structure was defined based on the AA models and maintained throughout the CG simulations. The CG proteins were then embedded into a lipid bilayer composed of C16:0/18:1 1-palmitoyl-2-oleoyl-phosphatidyl-choline (POPC), C16:0/18:1 1-palmitoyl-2-oleoyl-phosphatidyl-ethanolamine (POPE), C16:0/18:1 1-palmitoyl-2-oleoyl-phosphatidyl-glycerol (POPG), C16:0/18:1 1-palmitoyl-2-oleoyl-phosphatidyl-serine (POPS), C16:0/18:1 1-palmitoyl-2-oleoyl-phosphatidyl-inositol (POPI), C(d18:1/18:0) N-stearoyl-D-erythro-sphingomyelin (DPSM), and cholesterol (CHOL), at a molar ratio of 27:13:4:17:10:12:17. This lipid composition was used to reproduce the lipid composition of HEK cells, which were used for cryo-EM structural determination of prestin^18,52^. The initial orientation of prestin in the membrane was obtained from the OPM (Orientations of Proteins in Membranes) database^53^.

Four setups were constructed and simulated. In the first setup, we aimed to understand the impact of prestin on the overall deformation and lipid distribution of the membrane, for both contracted and expanded conformations. To enhance sampling of lipid-protein interactions and improve the statistics, in this setup which we refer to as the *quad-prestin* simulations, we placed four prestin dimers, separated by 200 Å, in a large patch of lipid bilayer (400 × 400 Å^2^), using INSANE^54^ (Fig. 1b).

In the second setup, we focused on the communication and cross-talk between the prestin dimers and how their individual deformation patterns might interfere with each other, and to compare their relative orientations. For this setup, referred to as *double-prestin* simulations, two prestin dimers, either in expanded or contracted conformations and separated by 200 Å, were placed in a rectangular lipid bilayer of dimensions 400 Å × 200 Å (Fig. 1c). To sample and compare multiple relative orientations, each prestin dimer was independently rotated around the *z* axis (membrane normal) at 45° intervals. Given the two-fold (C2) symmetry of prestin, placing each dimer at angles 0°, 45°, 90°, and 135° was sufficient to generate all possible 45° increments from 0 to 360° (Fig. 1d). To cover all the combinations of orientations of the two neighboring dimers, we constructed 16 different systems for each prestin’s conformational state (contracted or expanded) with the two protein dimers in one of the 4 different orientations: 4 orientations for Dimer I (0°, 45°, 90°, and 135°) × 4 orientations for Dimer II (0°, 45°, 90°, and 135°) = 16 systems. In these simulations, backbone restraints were used to maintain the positions and the relative orientations of the two dimers over the course of the simulations.

Using a third, extended simulation, we monitored free rotation of two neighboring dimers with respect to each other. Using the *double-prestin* setup described above, with the two protein dimers both initially at 90°, a simulation was performed for 40 μs during which only the centers of masses of the dimers were restrained (to prevent their translation), but they were allowed to freely rotate in their place. This simulation was used to sample the equilibrium distribution of the orientations.

Finally, to verify that our findings and conclusions regarding the preferred orientations of prestin are not affected by rectangular periodic boundary condition (PBC) used in Setups 2 and 3 (described above), we designed a forth simulation system containing nine copies of prestin dimers distanced 200 Å from each other and embedded in a lipid bilayer of 800 × 800 Å^2^ dimension (referred to as *nona-prestin* simulations; Supplementary Fig. 7). The center dimer was allowed to freely rotate with its center of mass restrained to avoid any translation, while the surrounding eight dimers were restrained both translationally and to a 45° orientation. This simulation set contained four independent systems with different initial orientations of the free-rotating prestin, to reduce the bias caused by the initial arrangement of the dimers.

All systems were then solvated and neutralized by counter ions, followed by ionization with 150 mM NaCl using INSANE^54^, before the simulations.

### Simulation protocol

All systems were simulated with GROMACS^55,56^, using the standard MARTINI v2.2 simulation settings^57^. A 20-fs timestep was employed, and the temperature was maintained at 310 K with the velocity-rescaling thermostat^58^ using a coupling time constant of 1 ps. A semi-isotropic, 1-bar pressure was maintained using the Berendsen barostat^59^ with a compressibility of 3 × 10^−4^ bar and a relaxation time constant of 5 ps. Initially, the systems were energy minimized for 1,000 steps, followed by short equilibration runs (18 ns) while lipid headgroups and protein backbones were restrained. Over this initial period, the restraints on lipid headgroups were gradually decreased in several steps from *k* = 200 kJ mol^−1^ nm^−2^ to zero, whereas the protein backbone restraints (*k* = 1000 kJ mol^−1^ nm^−2^) were maintained. For the production runs, the *quad-prestin* systems were simulated for 20 μs each (Supplementary Table 2). The 16 *double-prestin* systems with different orientations were simulated for 10 μs each (Supplementary Table 3). The third simulation system was initiated from the *double-prestin* system with both dimers at 90° (parallel) after 10 μs of equilibration. After removing the backbone restraints to allow the system to rotate freely, and introducing center of mass restraints for each prestin dimer to prevent their translation, the system was simulated for 40 μs. The *nona-prestin* systems (Setup 4) were each simulated for 5 μs (Supplementary Table 4).

### General analysis

VMD^51^ was used as a main analysis environment and to generate all the molecular images. In-house scripts were developed for specific geometrical analyses of the simulated systems, as described below. The position of a lipid or its distance to the protein was evaluated by the CG bead representing its phosphodiester PO_4_ in the case of phospholipids and sphingomyelin, or the hydroxy group in the case of cholesterol.

### Calculation of membrane deformation

The membrane deformation induced by prestin was evaluated by the height of the lipids (separation from the bilayer midplane, referred to as lipid elevation or depression) in each leaflet. The *z* position (along the membrane normal) of the phosphate beads (PO_4_ beads in MARTINI) of phospholipids was used for this purpose, with the bilayer midplane at *z* = 0. The data, averaged over the last 5 μs of each trajectory, were then binned in 2 × 2 Å^2^ bins to generate a 2-dimensional histogram in the *xy* plane (membrane plane). These histograms represent the average height (thickness) of each leaflet at different (*x*, *y*) coordinates. To quantify the propagation of membrane deformation, the maximum and minimum lipid heights were recorded at each distance (radius) with regard to the protein’s center in each frame, and their time-averaged values were plotted as a function of radius.

### Characterizing other membrane properties

To examine the health of the membrane systems, different membrane properties were monitored and analyzed throughout the quad-prestin simulations in both contracted and expanded prestin systems. These included: membrane thickness, total area, and atomic mass distribution of lipids and water along the membrane normal. The thickness of the membrane was calculated using the 2-dimensional histograms of lipid heights described earlier, and simply subtracting the lipid heights of outer leaflet from inner leaflet in each bin of the histogram. The total area of the membrane was readily calculated as the product of the *x* and *y* dimensions of the simulation box. The atomic mass distributions were calculated for the headgroup beads of phospholipids/sphingomyelin (phosphodiester group) and cholesterol (hydroxy group), as well as water beads. The distributions were histogrammed along the *z* axis and then their maximum population were scaled to one.

### Quantifying lipid distribution around prestin

To evaluate the effect of prestin on lipid distribution in the membrane, lipid counts within 7 Å of the protein were averaged for each lipid type over the 20 *μ*s of each *quad-prestin* simulation and compared to their bulk density. A depletion-enrichment index for lipid type L was then calculated as the ratio of the local and global fractions of the lipid^42^:

1 $${{{{{{{\rm{Depletion}}}}}}}}-{{{{{{{\rm{Enrichment}}}}}}}}\,{{{{{{{\rm{index}}}}}}}}\,({{{{{{{\rm{L}}}}}}}})=\frac{{f}_{{{{{{{{\rm{L}}}}}}}},7\AA }}{{f}_{{{{{{{{\rm{L}}}}}}}},{{{{{{{\rm{bulk}}}}}}}}}},$$

where

2 $${f}_{{{{{{{{\rm{L}}}}}}}},7\AA }=\frac{{{{{{{{\rm{Number}}}}}}}}\,{{{{{{{\rm{of}}}}}}}}\,{{{{{{{\rm{L}}}}}}}}\,{{{{{{{\rm{lipids}}}}}}}}\,{{{{{{{\rm{within}}}}}}}}\,7\,\AA \,{{{{{{{\rm{of}}}}}}}}\,{{{{{{{\rm{protein}}}}}}}}}{{{{{{{{\rm{Total}}}}}}}}\,{{{{{{{\rm{number}}}}}}}}\,{{{{{{{\rm{of}}}}}}}}\,{{{{{{{\rm{lipids}}}}}}}}\,{{{{{{{\rm{within}}}}}}}}\,7\,\AA \,{{{{{{{\rm{of}}}}}}}}\,{{{{{{{\rm{the}}}}}}}}\,{{{{{{{\rm{protein}}}}}}}}}$$

and

3 $${f}_{{{{{{{{\rm{L,bulk}}}}}}}}}=\frac{{{{{{{{\rm{Total}}}}}}}}\,{{{{{{{\rm{number}}}}}}}}\,{{{{{{{\rm{of}}}}}}}}\,{{{{{{{\rm{L}}}}}}}}\,{{{{{{{\rm{lipids}}}}}}}}\,{{{{{{{\rm{in}}}}}}}}\,{{{{{{{\rm{the}}}}}}}}\,{{{{{{{\rm{membrane}}}}}}}}}{{{{{{{{\rm{Total}}}}}}}}\,{{{{{{{\rm{number}}}}}}}}\,{{{{{{{\rm{of}}}}}}}}\,{{{{{{{\rm{lipids}}}}}}}}\,{{{{{{{\rm{in}}}}}}}}\,{{{{{{{\rm{the}}}}}}}}\,{{{{{{{\rm{membrane}}}}}}}}}$$

### Estimation of membrane bending modulus

To calculate the bending modulus, we applied the Helfrich-Canham (HC) theory, which relates the equilibrium fluctuations of a membrane to its elastic properties^60–62^. Even though the HC theory is best used in membranes without significant deformations, it provides a way to quantitatively estimate and compare the mechanical properties of the membrane in response to different arrangements of prestin. In order to reduce the effect of the lipids in the immediate vicinity of the protein, which are largely deformed, we limited this analysis to the lipids that are at least 7 Å away from the protein. The fluctuations along the the *z* direction were calculated for the phosphate bead (PO_4_) of each phospholipid and then histogrammed over the membrane (*xy*) plane using 1 × 1 Å^2^ bins. The fluctuations were then transformed to the Fourier space and employed in the following equation to calculate the bending modulus^63^:

4 $$|h(q){|}^{2}=\frac{{k}_{{{{{{{{\rm{B}}}}}}}}}T}{K{q}^{4}+\sigma {q}^{2}},$$

where *h*(*q*) is the fluctuation spectrum in the *z* direction in the Fourier space, *k*_B_ is the Boltzmann constant, *T* is the temperature, *K* is the bending modulus, *σ* is the surface tension, and *q* is the magnitude of the wavevector (i.e., the wavenumber)^63^. To obtain the bending modulus, ∣*h*(*q*)∣^2^ (referred to as intensity) was plotted with respect to *q* in a log-log format, yielding two different regions: (1) the region with free undulation, where the effect of the macroscopic protrusion is minimal, fitted to *q*^−4^, and, (2) a region in which the effect of protrusion is expected to be larger, and fitted to *q*^−2^. The y-intercept of a line fitted to the free-undulation region on the log-log plot corresponds to $\frac{{k}_{{{{{{{{\rm{B}}}}}}}}}T}{K}$ and can be used to calculate the bending modulus. All the *K* values were divided by the maximum *K* for each prestin conformation (expanded or contracted), generating scaled bending moduli, which were used for comparing the systems.

### Reporting summary

Further information on research design is available in the Nature Portfolio Reporting Summary linked to this article.

## Supplementary information

Supplementary Information

Description of Additional Supplementary Files

Supplementary Movie 1

Supplementary Movie 2

Reporting Summary

## Data Availability

MD trajectories, parameter files, and input files have been deposited in an open public repository [10.5281/zenodo.7028021]. The cryo-EM structure mentioned in the study is available under the PDB accession code 7LGW [10.2210/pdb7LGW/pdb]. Source data are provided with this paper.

## Source data

Source Data

## Code availability

All simulation were performed using a publicly available software suite for high-performance MD simulations, GROMACS v2020.3 [https://www.gromacs.org/] and analyzed using VMD v1.9.4 [https://www.ks.uiuc.edu/Research/vmd/], as well as in-house scripts. In-house scripts employed to analyze the MD trajectories are available in an open public repository [10.5281/zenodo.7028021].
