# Supplementary material for: Lipid-mediated prestin organization in outer hair cell membranes and its implications in sound amplification
Source: Nat Commun. 2022 Nov 12;13:6877. doi: 10.1038/s41467-022-34596-9 (PMC9653410; doi:10.1038/s41467-022-34596-9)
Supplement: Supplementary file 1 — Supplementary Information [file 41467_2022_34596_MOESM1_ESM.pdf]

SUPPLEMENTARY INFORMATION

# Lipid-mediated prestin organization in outer hair cell membranes and its implications in sound amplification

Sepehr Dehghani-Ghahnaviyeh, Zhiyu Zhao, and Emad Tajkhorshid*

Theoretical and Computational Biophysics Group, NIH Center for Macromolecular Modeling and Bioinformatics, Beckman Institute for Advanced Science and Technology, Department of Biochemistry, and Center for Biophysics and Quantitative Biology, University of Illinois at Urbana-Champaign, Urbana, IL, USA

* To whom correspondence should be addressed; email: emad@illinois.edu

Supplementary Table 1: **Bending moduli values.** Bending moduli, $K$, are calculated for contracted and expanded *double-prestin* systems with different arrangements of protein dimers. The last column represents the mean square error (MSE) for the fitted line to the free-undulation region to calculate $K$.

| **Conformation** | **Orientation of Dimer I** | **Orientation of Dimer II** | **K (kcal/mol)** | **MSE (kcal/mol)$^2$** |
|---|---|---|---|---|
| Contracted | 0 | 0 | 63.21 | 0.48 |
| Contracted | 0 | 45 | 45.01 | 0.181 |
| Contracted | 0 | 90 | 47.85 | 0.41 |
| Contracted | 0 | 135 | 37.51 | 0.05 |
| Contracted | 45 | 0 | 45.61 | 0.17 |
| Contracted | 45 | 45 | 22.19 | 0.16 |
| Contracted | 45 | 90 | 39.24 | 0.03 |
| Contracted | 45 | 135 | 50.85 | 0.31 |
| Contracted | 90 | 0 | 49.86 | 0.35 |
| Contracted | 90 | 45 | 38.99 | 0.04 |
| Contracted | 90 | 90 | 65.32 | 0.34 |
| Contracted | 90 | 135 | 49.49 | 0.09 |
| Contracted | 135 | 0 | 37.51 | 0.05 |
| Contracted | 135 | 45 | 49.68 | 0.30 |
| Contracted | 135 | 90 | 42.53 | 0.07 |
| Contracted | 135 | 135 | 27.51 | 0.15 |
| Expanded | 0 | 0 | 70.28 | 0.19 |
| Expanded | 0 | 45 | 50.10 | 0.11 |
| Expanded | 0 | 90 | 59.37 | 0.19 |
| Expanded | 0 | 135 | 46.07 | 0.05 |
| Expanded | 45 | 0 | 49.73 | 0.10 |
| Expanded | 45 | 45 | 28.02 | 0.14 |
| Expanded | 45 | 90 | 43.84 | 0.02 |
| Expanded | 45 | 135 | 57.77 | 0.18 |
| Expanded | 90 | 0 | 64.83 | 0.19 |
| Expanded | 90 | 45 | 45.53 | 0.05 |
| Expanded | 90 | 90 | 77.11 | 0.22 |
| Expanded | 90 | 135 | 57.52 | 0.06 |
| Expanded | 135 | 0 | 47.17 | 0.06 |
| Expanded | 135 | 45 | 65.02 | 0.21 |
| Expanded | 135 | 90 | 56.85 | 0.07 |
| Expanded | 135 | 135 | 25.64 | 0.22 |

Supplementary Table 2: **Quad-prestin simulations.** Each system included four copies of prestin dimer arranged in a square configuration with a side length of 100 Å and simulated for 20 $\mu$s, resulting in a collective sampling time of 80 $\mu$s (4 dimers × 20 $\mu$s = 80 $\mu$s) of lipid-protein interactions per prestin conformation (contracted or expanded) and an overall simulation time of 160 $\mu$s.

| System | Conformation | Number of dimers | Simulation time per dimer | Total sampling time |
|---|---|---|---|---|
| 1 | Contracted | 4 | 20 $\mu$s | 80 $\mu$s |
| 2 | Expanded | 4 | 20 $\mu$s | 80 $\mu$s |

Supplementary Table 3: **Double-prestin simulations.** Each system included a pair of prestin dimers, either in contracted on in expanded conformations, in one of the 16 different relative orientations between the two dimers, and simulated for 10 $\mu$s, resulting collectively in 320 $\mu$s (16 orientations $\times$ 2 conformations $\times$ 10 $\mu$s = 320 $\mu$s) of simulation time.

| System | Conformation | Orientation of Dimer I | Orientation of Dimer II | Simulation time |
|---|---|---|---|---|
| 1 | Contracted | 0 | 0 | 10 $\mu$s |
| 2 | Contracted | 0 | 45 | 10 $\mu$s |
| 3 | Contracted | 0 | 90 | 10 $\mu$s |
| 4 | Contracted | 0 | 135 | 10 $\mu$s |
| 5 | Contracted | 45 | 0 | 10 $\mu$s |
| 6 | Contracted | 45 | 45 | 10 $\mu$s |
| 7 | Contracted | 45 | 90 | 10 $\mu$s |
| 8 | Contracted | 45 | 135 | 10 $\mu$s |
| 9 | Contracted | 90 | 0 | 10 $\mu$s |
| 10 | Contracted | 90 | 45 | 10 $\mu$s |
| 11 | Contracted | 90 | 90 | 10 $\mu$s |
| 12 | Contracted | 90 | 135 | 10 $\mu$s |
| 13 | Contracted | 135 | 0 | 10 $\mu$s |
| 14 | Contracted | 135 | 45 | 10 $\mu$s |
| 15 | Contracted | 135 | 90 | 10 $\mu$s |
| 16 | Contracted | 135 | 135 | 10 $\mu$s |
| 17 | Expanded | 0 | 0 | 10 $\mu$s |
| 18 | Expanded | 0 | 45 | 10 $\mu$s |
| 19 | Expanded | 0 | 90 | 10 $\mu$s |
| 20 | Expanded | 0 | 135 | 10 $\mu$s |
| 21 | Expanded | 45 | 0 | 10 $\mu$s |
| 22 | Expanded | 45 | 45 | 10 $\mu$s |
| 23 | Expanded | 45 | 90 | 10 $\mu$s |
| 24 | Expanded | 45 | 135 | 10 $\mu$s |
| 25 | Expanded | 90 | 0 | 10 $\mu$s |
| 26 | Expanded | 90 | 45 | 10 $\mu$s |
| 27 | Expanded | 90 | 90 | 10 $\mu$s |
| 28 | Expanded | 90 | 135 | 10 $\mu$s |
| 29 | Expanded | 135 | 0 | 10 $\mu$s |
| 30 | Expanded | 135 | 45 | 10 $\mu$s |
| 31 | Expanded | 135 | 90 | 10 $\mu$s |
| 32 | Expanded | 135 | 135 | 10 $\mu$s |

Supplementary Table 4: **Nona-prestin simulations.** Each system included nine copies of prestin dimers arranged in a square array with a side length of 800 Å and simulated for 5 $\mu$s. Different simulations started from different orientations of free-rotating prestin dimer, centered in the simulation box.

| System | Number of dimers | Initial orientation of free-rotating prestin | Simulation time |
|---|---|---|---|
| 1 | 9 | 0° | 5 $\mu$s |
| 2 | 9 | 45° | 5 $\mu$s |
| 3 | 9 | 90° | 5 $\mu$s |
| 4 | 9 | 135° | 5 $\mu$s |

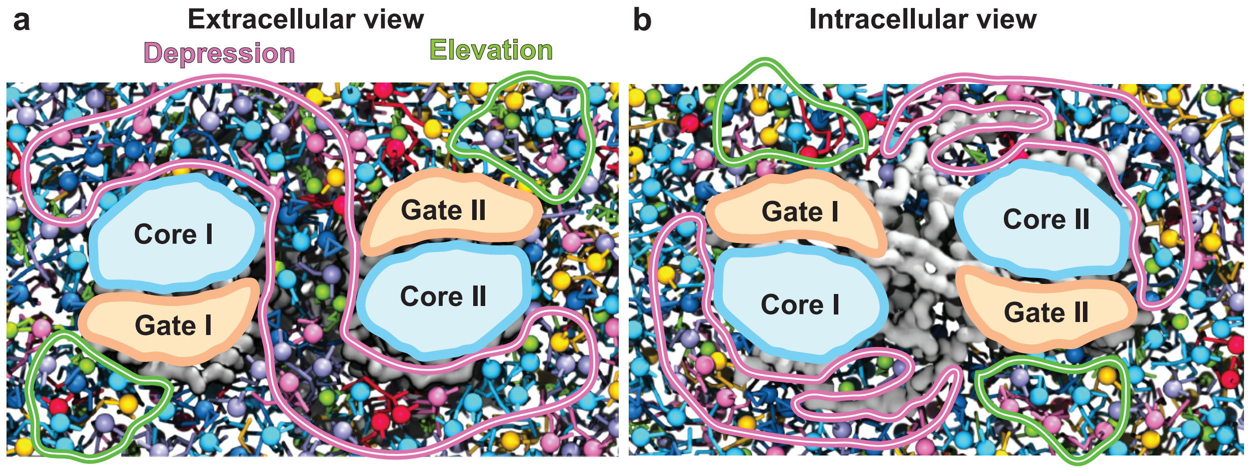


Supplementary Figure 1: **Membrane deformation pattern around prestin.** Extracellular **(a)** and intracellular **(b)** views of a membrane-embedded prestin dimer after 20 $\mu$s of simulation. Regions with significant lipid elevation (green) or depression (pink) are highlighted in the vicinity of the gate (orange) and core (blue) domains, respectively. PC, PI, SM, CHOL, PS, PG, and PE lipids are shown in cyan, blue, magenta, green, purple, red, and yellow, respectively.

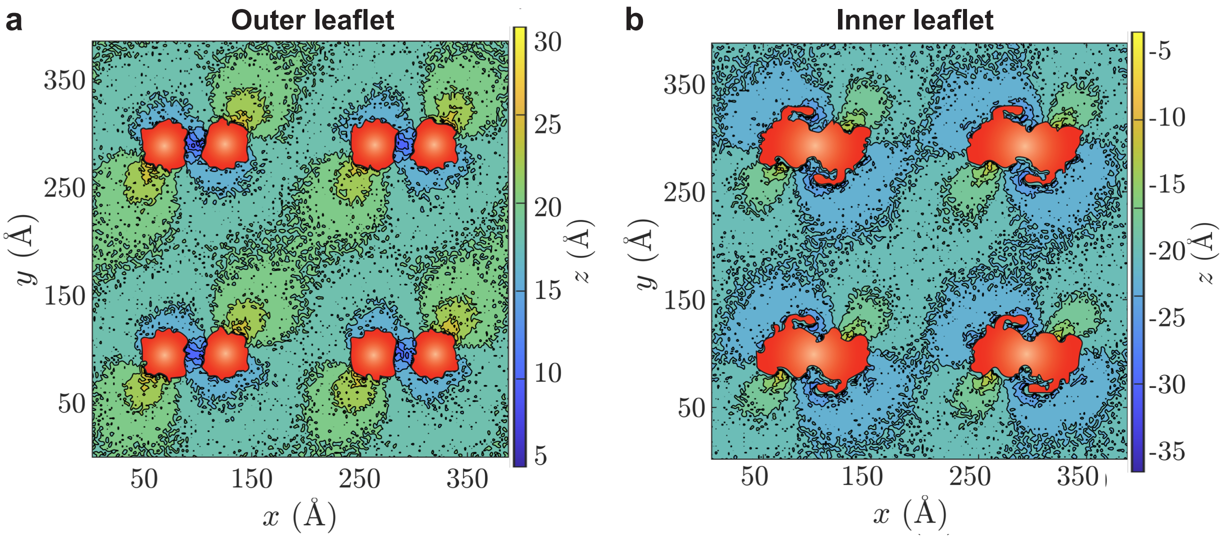


Supplementary Figure 2: **Prestin-induced, anisotropic membrane deformation for the expanded conformation.** 2D histograms of the $z$ positions of lipid headgroups for the outer **(a)** and inner **(b)** leaflets. Both leaflets are viewed from the extracellular side. Histograms are constructed from the last 5 $\mu$s of a 20-$\mu$s trajectory. The cross sectional areas of the proteins in each leaflet are drawn in red.

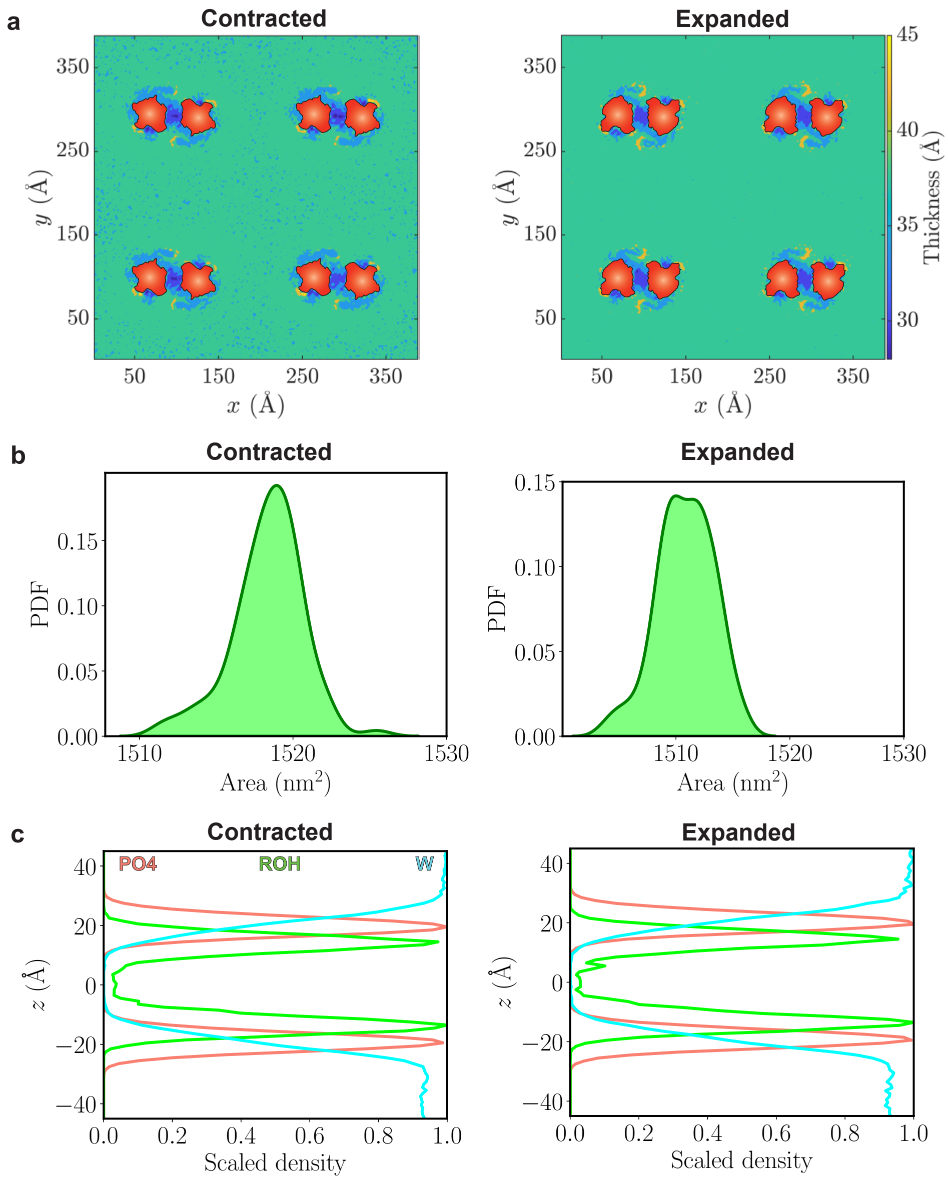


Supplementary Figure 3: **Different properties of the lipid bilayer for the quad-prestin simulation systems. (a)** Lipid bilayer thickness calculated for contracted and expanded quad-prestin systems. The cross section of the protein on the cytoplasmic and extracellular sides are shown in red. **(b)** Histograms representing the total area of the lipid bilayer for simulations with contracted or expanded prestin systems. The *y* axis is probability distribution function (PDF). **(c)** Atomic mass distribution for lipid headgroups (phosphate beads, $PO_4$ for phospholipids and hydroxy beads, ROH, for cholesterol), as well as for water beads (W).

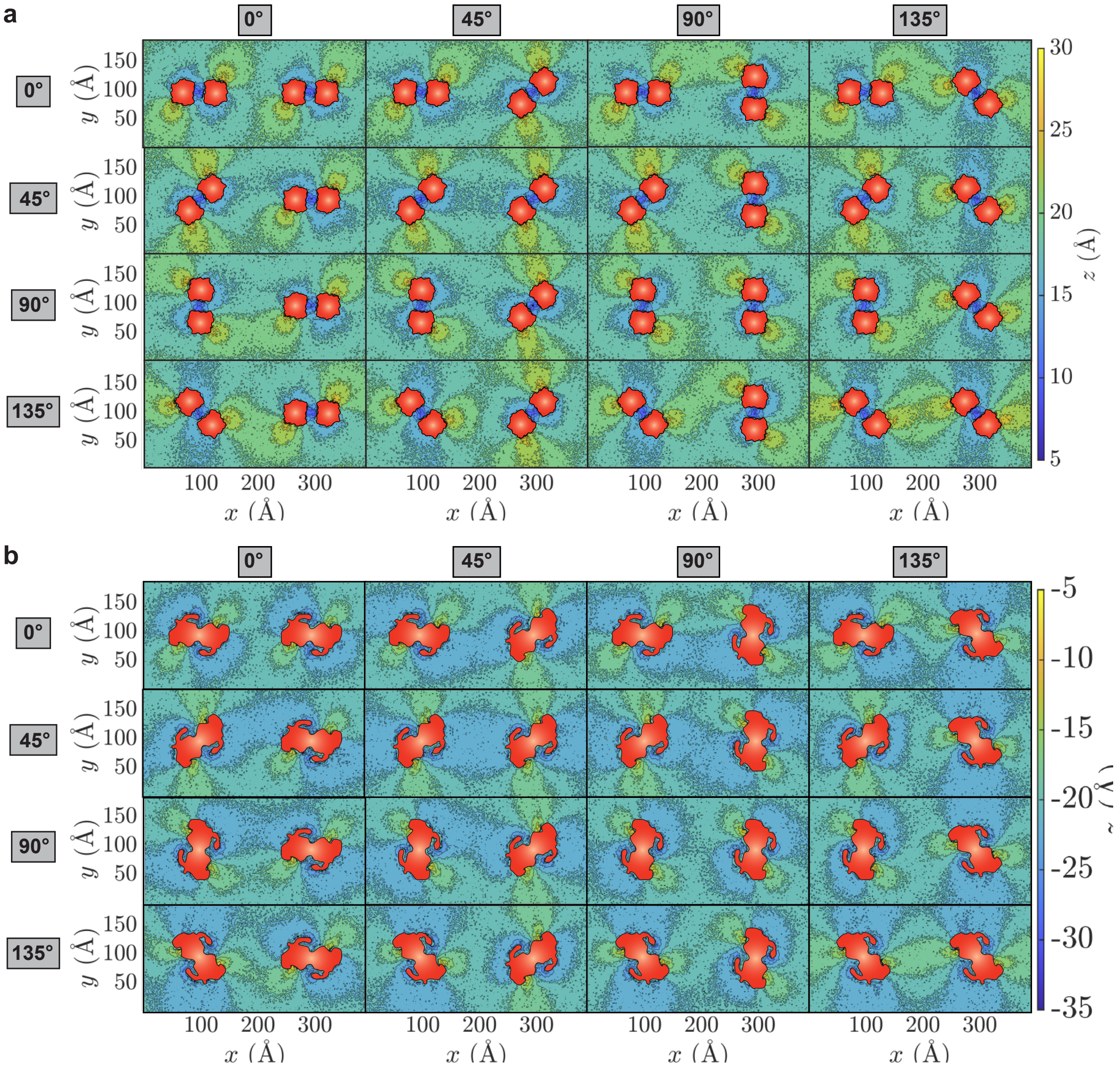


Supplementary Figure 4: **Constructive or destructive interference of deformation patterns induced by expanded prestin's different orientations**. Heatmaps of phospholipid height in the outer **(a)** and inner **(b)** leaflets are calculated using the procedure described in Fig. 2. The prestin dimers are placed at $(x, y) = (100, 100)$ Å and $(300, 100)$ Å, respectively. The orientation of Dimer I (angle with respect to the $x$ axis) is specified on the left, and for Dimer II on the top of the panel. The protein cross sectional area in each leaflet is drawn in red.

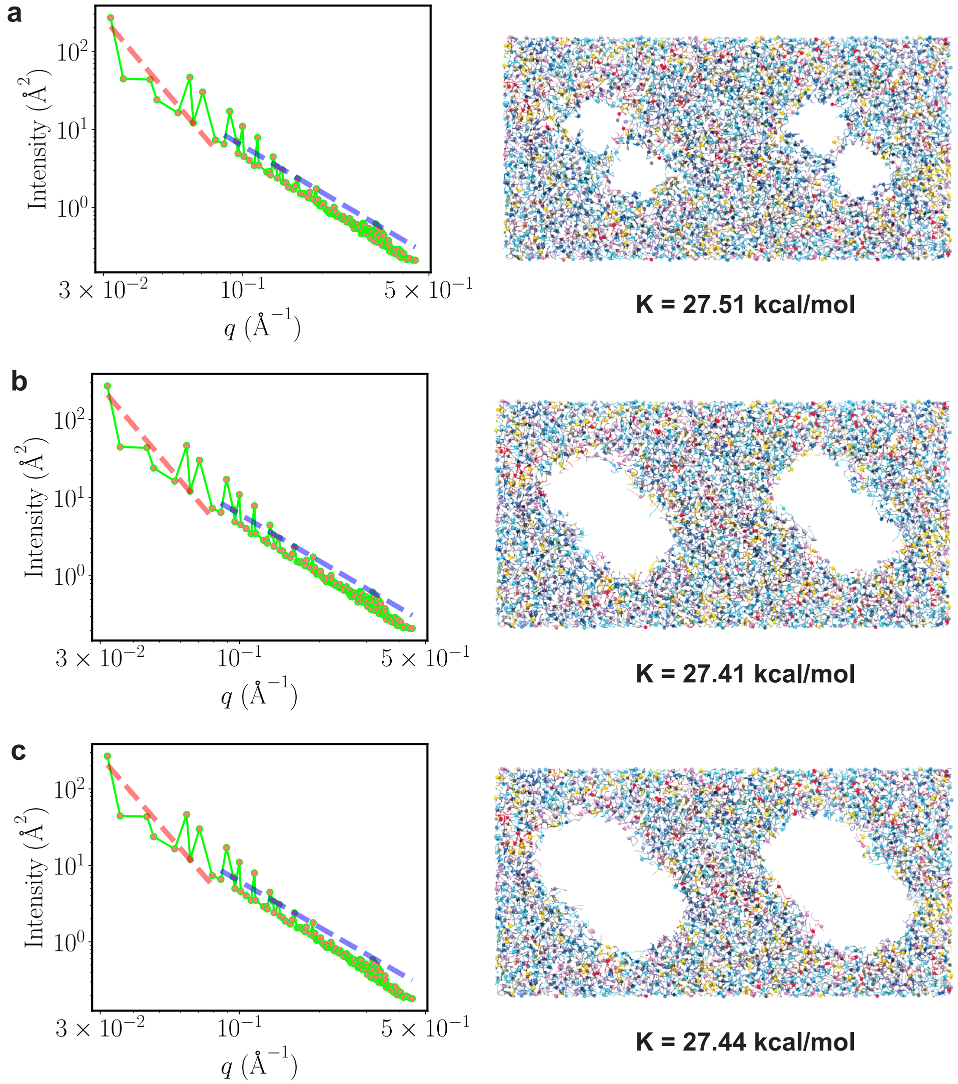


Supplementary Figure 5: **Bending modulus calculations with different selections of lipids.** **(a)** Bending modulus calculation including the entire lipid bilayer for the [135°, 135°] system. **(b)** and **(c)** Bending modulus calculations for the same system as in **a** after excluding lipids within 7 Å (b) or 14 Å (c). Log-log plots of intensity vs. $q$, include free-undulation (fitted to a red line) and protrusion (fitted to a blue line) regions. The fitted red line was employed to obtain the bending moduli. Right panels show the lipid selection in each case. The three selections result very similar bending moduli (within 0.1 kcal/mol). Source data are provided as a Source Data file.

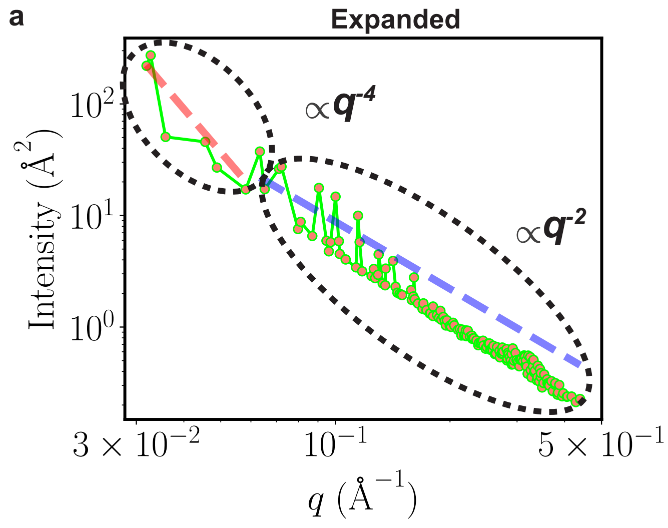


Supplementary Figure 6: **Membrane bending moduli calculated for different expanded prestin configurations**. **(a)** Intensity, $|h(q)|^2$, plotted vs. $q$, wavenumber, for the system with two prestin dimers both at 135° ([135°, 135°]). The plot is in log-log scale and contains a free-undulation region fitted by $q^{-4}$ line (shown in red) and a protrusion region fitted by $q^{-2}$ line (shown in blue). The fit to the free-undulation region is employed to calculate the bending modulus. Source data are provided as a Source Data file. **(b)** Bending moduli for the membranes with two prestin dimers in different orientations (Fig. 3), normalized by the maximum value for the [45°, 135°] system. The orientation of Dimer 1, placed at $(x, y) = (100, 100)$ Å is shown on the left, and for Dimer II, placed at $(x, y) = (300, 100)$ Å, on the top of the table. The color shade in each box also represents the strength of the bending modulus, and shown in the scale bar. The minimum bending moduli are captured for the system with two prestin dimers at either 45° or 135°.

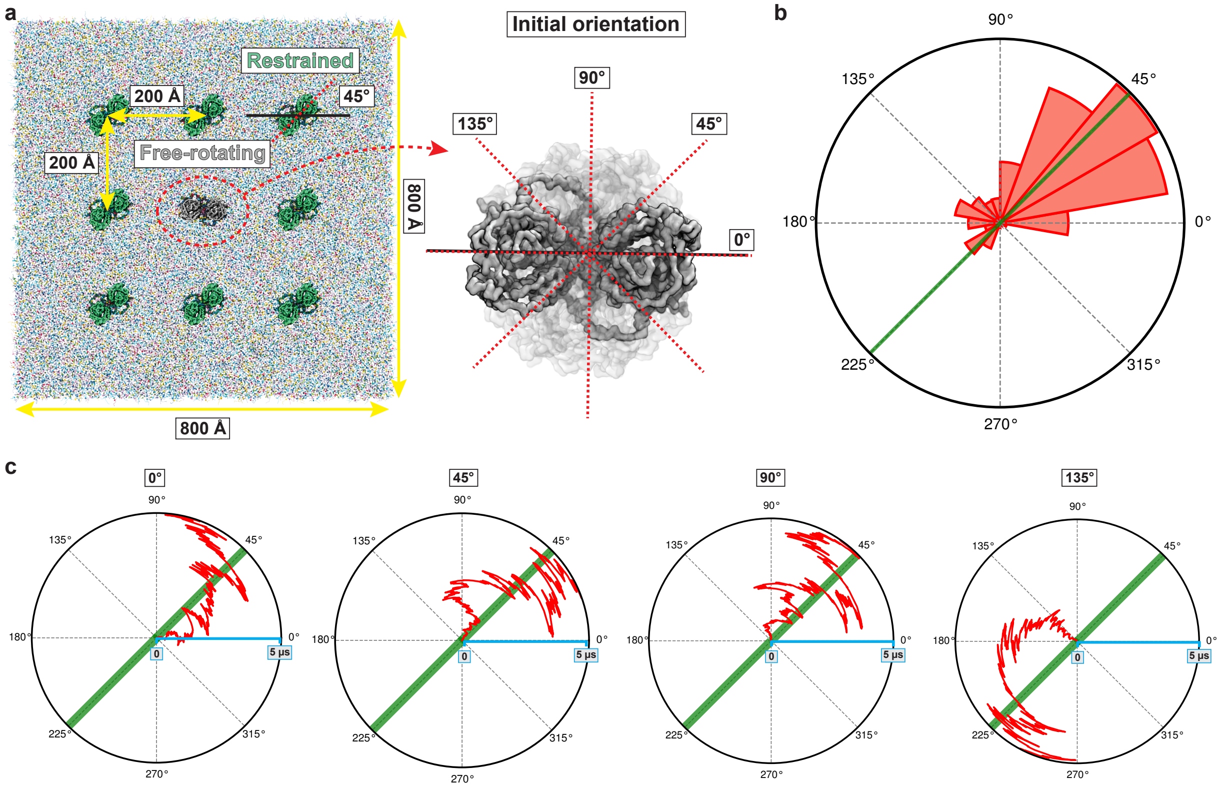


Supplementary Figure 7: **Examining the effect of PBC. (a)** Initial system setup containing nine prestin dimers distanced 200 Å with respect to each other and placed in a lipid bilayer with 800 Å × 800 Å dimension (nona-prestin system). The centered dimer (shown in gray) is free to rotate, whereas the others (shown in green) are restrained to 45°. Four different systems are simulated in this set with the free-rotating prestin initially placed at different orientations (0°, 45°, 90°, and 135°). **(b)** Orientation of the free-rotating dimer, histogrammed over the last 4 $\mu$s of trajectories. The maximum population of the histogram is located around 45° (highlighted in green), indicating that the prestin arrangement predicted here is not affected by PBC. **(c)** Time series demonstrating the orientation of free-rotating prestin throughout the 5 $\mu$s of simulations for all four systems. The free-rotating dimer in all the simulations fluctuates around 45°/225° (highlighted in green). Source data are provided as a Source Data file.

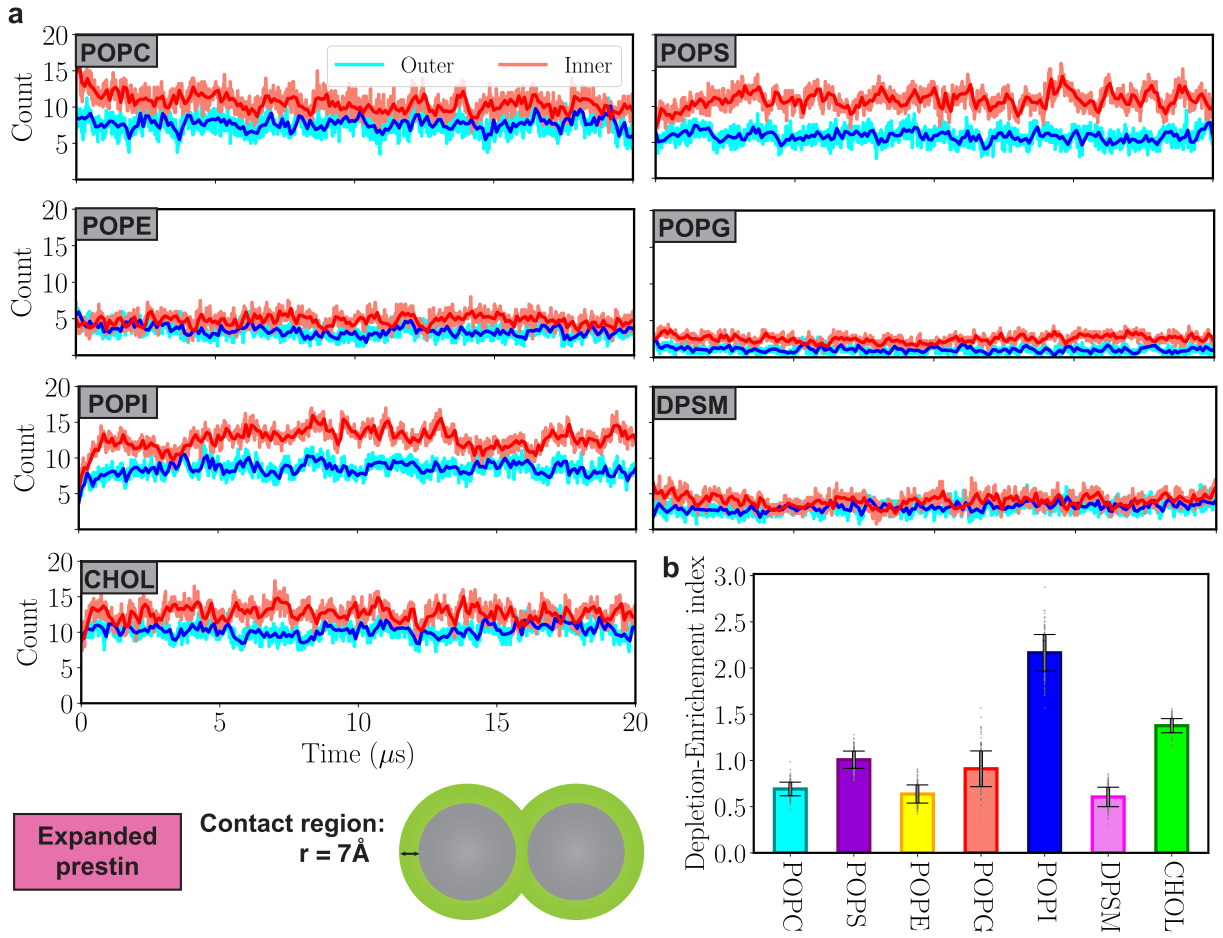


Supplementary Figure 8: **Enrichment/depletion of different lipids around expanded prestin**. **(a)** Time series of the number of different lipid types within 7 Å of prestin, averaged over four dimers, in the outer (blue) and inner (red) leaflets. PI lipids accumulate the most around prestin in the inner leaflet. **(b)** Depletion-Enrichment index of each lipid type (the ratio of the local and bulk fractions of the lipid). PI and CHOL show the highest enrichment, whereas SM and PE show largest extents of depletion. Data points in each bar plot are shown as gray dots. Error bars are standard deviation of the data obtained from 2000 data points. Source data are provided as a Source Data file.

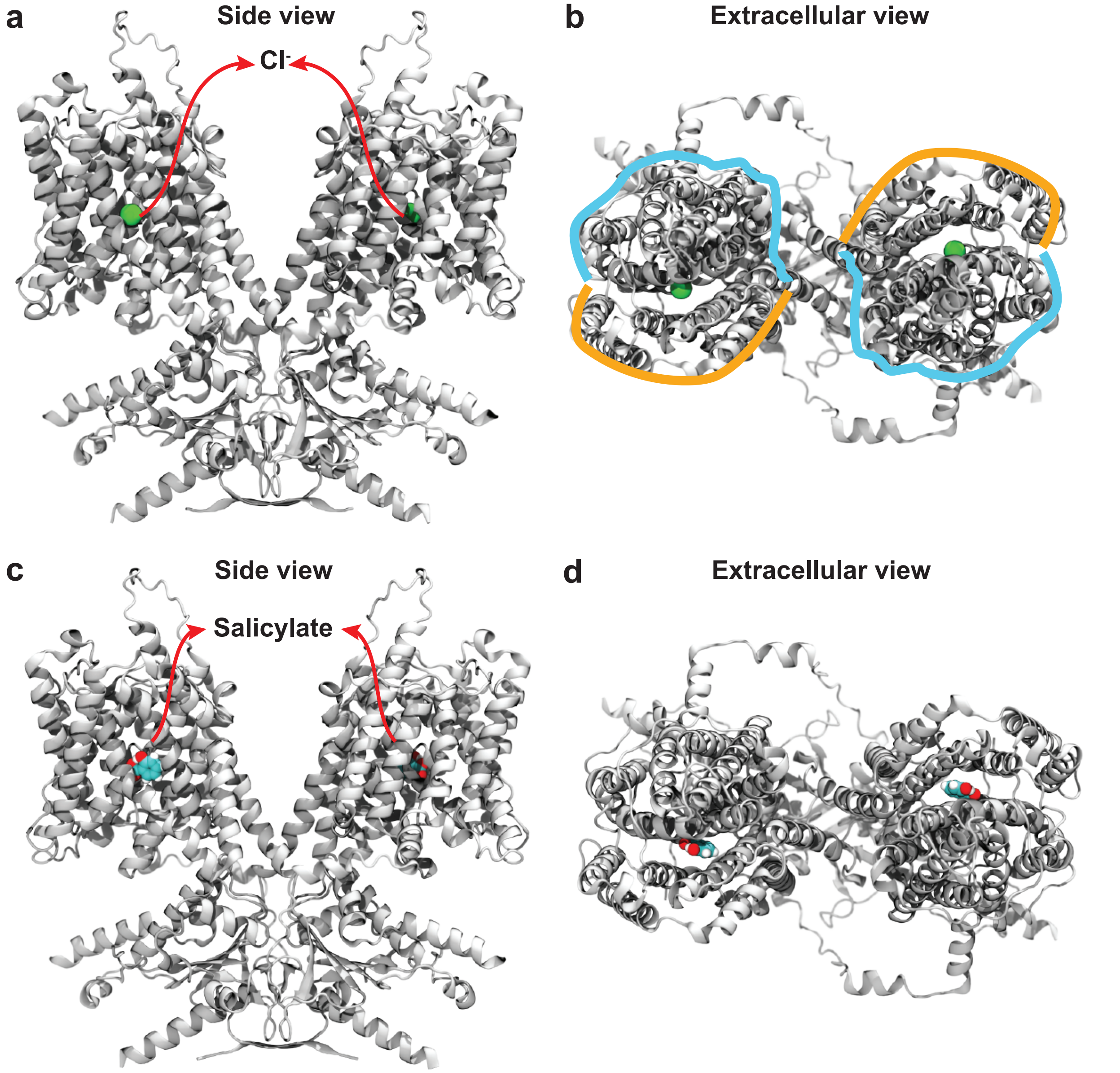


Supplementary Figure 9: **All-atom models of prestin in different functional (conformational) states.** **(a and b)** Side and extracellular views of contracted prestin bound to $Cl^-$ (green). The core and gate domains are highlighted in blue and orange, respectively. **(c and d)** Membrane and extracellular views of expanded prestin bound to the inhibitor salicylate. Both ligands, $Cl^-$ and salicylate, bind to the same site, located in between the core and gate domains. The contracted conformation has a smaller cross-sectional area in the membrane.
