# Supplementary material for: Lipid-mediated prestin organization in outer hair cell membranes and its implications in sound amplification
Source: Nat Commun. 2022 Nov 12;13:6877. doi: 10.1038/s41467-022-34596-9 (PMC9653410; doi:10.1038/s41467-022-34596-9)
Supplement: Supplementary file 2 — Description of Additional Supplementary Files [file 41467_2022_34596_MOESM2_ESM.docx]

**Description of Additional Supplementary Files**

**Supplementary Movie 1**. Comparison of the membrane deformation obtained from the simulations (colored lipids) with that from the CryoEM electron density (red mesh) of prestin in nanodiscs.

**Supplementary Movie 2**. 40 𝜇s trajectory highlighting through-lipid communication of neighboring prestin dimers. The orientation of the two dimers is tracked along time, and the populations of the tracked angles are plotted as a heat map.
