# Supplementary material for: Lipid-mediated prestin organization in outer hair cell membranes and its implications in sound amplification
Source: Nat Commun. 2022 Nov 12;13:6877. doi: 10.1038/s41467-022-34596-9 (PMC9653410; doi:10.1038/s41467-022-34596-9)
Supplement: Supplementary file 5 — Reporting Summary [file 41467_2022_34596_MOESM5_ESM.pdf]

nature portfolio

Corresponding author(s): Emad Tajkhorshid

Last updated by author(s): 10/16/2022

Reporting Summary

Nature Portfolio wishes to improve the reproducibility of the work that we publish. This form provides structure for consistency and transparency in reporting. For further information on Nature Portfolio policies, see our Editorial Policies and the Editorial Policy Checklist.

Statistics

For all statistical analyses, confirm that the following items are present in the figure legend, table legend, main text, or Methods section.

n/a	Confirmed	
☐	☒	The exact sample size (*n*) for each experimental group/condition, given as a discrete number and unit of measurement
☒	☐	A statement on whether measurements were taken from distinct samples or whether the same sample was measured repeatedly
☒	☐	The statistical test(s) used AND whether they are one- or two-sided<br>*Only common tests should be described solely by name; describe more complex techniques in the Methods section.*
☒	☐	A description of all covariates tested
☒	☐	A description of any assumptions or corrections, such as tests of normality and adjustment for multiple comparisons
☐	☒	A full description of the statistical parameters including central tendency (e.g. means) or other basic estimates (e.g. regression coefficient) AND variation (e.g. standard deviation) or associated estimates of uncertainty (e.g. confidence intervals)
☒	☐	For null hypothesis testing, the test statistic (e.g. *F*, *t*, *r*) with confidence intervals, effect sizes, degrees of freedom and *P* value noted<br>*Give P values as exact values whenever suitable.*
☒	☐	For Bayesian analysis, information on the choice of priors and Markov chain Monte Carlo settings
☒	☐	For hierarchical and complex designs, identification of the appropriate level for tests and full reporting of outcomes
☒	☐	Estimates of effect sizes (e.g. Cohen's *d*, Pearson's *r*), indicating how they were calculated

Our web collection on statistics for biologists contains articles on many of the points above.

Software and code

Policy information about availability of computer code

Data collection	Publicly available software package GROMACS v2020.3 was used for the simulations (https://www.gromacs.org).
Data analysis	Publicly available software package VMD v1.9.4 was used for the analysis and visualization (https://www.ks.uiuc.edu/Research/vmd/). In-house scripts employed to analyse the MD trajectories are available in an open public repository: https://doi.org/10.5281/zenodo.7028021

For manuscripts utilizing custom algorithms or software that are central to the research but not yet described in published literature, software must be made available to editors and reviewers. We strongly encourage code deposition in a community repository (e.g. GitHub). See the Nature Portfolio guidelines for submitting code & software for further information.

Data

Policy information about availability of data

All manuscripts must include a data availability statement. This statement should provide the following information, where applicable:

- Accession codes, unique identifiers, or web links for publicly available datasets
- A description of any restrictions on data availability
- For clinical datasets or third party data, please ensure that the statement adheres to our policy

MD trajectories, parameter files, and input files have been deposited in an open public repository [https://doi.org/10.5281/zenodo.7028021].

Human research participants

Policy information about studies involving human research participants and Sex and Gender in Research.

Reporting on sex and gender	Not applicable to our study.
Population characteristics	Not applicable to our study.
Recruitment	Not applicable to our study.
Ethics oversight	Not applicable to our study.

Note that full information on the approval of the study protocol must also be provided in the manuscript.

Field-specific reporting

Please select the one below that is the best fit for your research. If you are not sure, read the appropriate sections before making your selection.

☒ Life sciences ☐ Behavioural & social sciences ☐ Ecological, evolutionary & environmental sciences

For a reference copy of the document with all sections, see nature.com/documents/nr-reporting-summary-flat.pdf

Life sciences study design

All studies must disclose on these points even when the disclosure is negative.

Sample size	When applicable, data sample size was determined based on previous reports in the literature.<br>see, e.g., https://pubs.acs.org/doi/full/10.1021/acscentsci.8b00143 and https://pubs.rsc.org/en/content/articlehtml/2016/sm/c6sm01186a
Data exclusions	No data were excluded from the analyses.
Replication	Simulation results are based on multiple independent copies. Membrane deformation patterns were confirmed by four independent simulation replicas, all yielding the same deformation patterns.
Randomization	All the membrane systems were independently generated initially based on a random model generator.
Blinding	Not applicable to our study. The study does not include samples from individuals that could be subject to blinding.

Reporting for specific materials, systems and methods

We require information from authors about some types of materials, experimental systems and methods used in many studies. Here, indicate whether each material, system or method listed is relevant to your study. If you are not sure if a list item applies to your research, read the appropriate section before selecting a response.

Materials & experimental systems

n/a	Involved in the study	
☒	☐	Antibodies
☒	☐	Eukaryotic cell lines
☒	☐	Palaeontology and archaeology
☒	☐	Animals and other organisms
☒	☐	Clinical data
☒	☐	Dual use research of concern

Methods

n/a	Involved in the study	
☒	☐	ChIP-seq
☒	☐	Flow cytometry
☒	☐	MRI-based neuroimaging
